# Cancer Treatment Dosing Regimens of Zoledronic Acid Result in Near-Complete Suppression of Mandible Intracortical Bone Remodeling in Beagle Dogs

**DOI:** 10.1359/jbmr.090713

**Published:** 2009-07-06

**Authors:** Matthew R Allen, Daniel J Kubek, David B Burr

**Affiliations:** 1 Department of Anatomy and Cell Biology Indianapolis, IN, USA; 2 Department of Orthopaedic Surgery, Indiana University School of Medicine Indianapolis, IN, USA; 3 Department of Biomedical Engineering Program, Indiana University–Purdue University Indianapolis Indianapolis, IN, USA

**Keywords:** bisphosphonates, cancer treatment, jaw, remodeling suppression, osteonecrosis

## Abstract

Bisphosphonate doses used in cancer treatment are substantially higher than those used for osteoporosis. Little is known about the effects of these high doses on tissue-level remodeling suppression. The aim of this study was to assess the effects of cancer dosing regimens of zoledronic acid on tissue-level bone remodeling at different skeletal sites. Skeletally mature female beagle dogs were treated with monthly intravenous infusions of vehicle (VEH, saline) or zoledronic acid (ZOL, 0.067 mg/kg); an additional group of animals was treated daily with oral alendronate (ALN, 0.2 mg/kg/day). Doses of ZOL and ALN were, on a milligram per kilogram basis, consistent with those used for cancer and osteoporosis, respectively. Following either 3 or 6 months of treatment, animals were euthanized, and mandible, rib, and tibia were processed for dynamic bone histology. There was no evidence of oral lesions or bone matrix necrosis in the mandibles of any animals. After 3 months, the rate of intracortical bone remodeling in the mandible was significantly suppressed with ZOL (−95%) compared with VEH; by 6 months, ZOL had produced nearly complete suppression (−99%) compared with VEH. ZOL also significantly suppressed remodeling in the rib cortex at both 3 (−83%) and 6 (−85%) months compared with VEH; tibia cortex bone formation rate was nonsignificantly lower with ZOL treatment (−68% to −75%). Remodeling suppression in ZOL-treated animals was significantly greater than in ALN-treated animals at both the mandible and the rib; ALN and VEH were not different for any of the assessed parameters at any of the sites. Compared across skeletal sites, the absolute level of remodeling suppression with ZOL treatment was significantly greater at sites with higher remodeling, whereas the percent reduction was similar among the sites. These results document nearly complete intracortical remodeling suppression resulting from monthly intravenous zoledronic acid dosing, with changes being most dramatic at the mandible. Copyright © 2010 American Society for Bone and Mineral Research

## Introduction

Since first approved for treating malignant hypercalcemia, bisphosphonates (BPs) have emerged as the gold standard treatment for a number of metabolic bone diseases. The overwhelming majority of BP prescriptions are written for postmenopausal osteoporosis,(1) yet BPs have become a standard component of treatment/prevention for malignant hypercalcemia and bone metastases in cancer patients.(2) In all these clinical settings, BPs exert their skeletal effect by reducing bone remodeling.(1,3,4)

The emergence of bisphosphonate-related osteonecrosis of the jaw (BRONJ) has raised concern about BPs, although a clear cause-and-effect relationship has yet to be established between BPs and BRONJ.(5) Most BRONJ cases have manifested in patients administered high doses of intravenous BPs for treatment/prevention of cancer-related malignancies,(6–9) with a smaller number of cases reported in patients receiving oral BPs for treatment of postmenopausal osteoporosis.(5,9,10) While the underlying pathophysiology of BRONJ remains unclear, most hypotheses implicate remodeling suppression as an underlying tissue-level mechanism(11–14) because remodeling rates of the mandible have been shown to exceed those of other cortical bone sites.(15–17)

Serum/urine biomarkers, measures of systemic bone remodeling, have been studied in several cohorts of cancer patients treated with BPs.(18) These studies have shown that zoledronic acid, administered at a dose of 4 mg as an intravenous infusion, significantly reduces bone remodeling by up to 80%.(19–22) While no head-to-head comparison exists, these systemic levels of remodeling suppression with cancer doses of zoledronic acid are similar in magnitude to those achieved with BP doses used for osteoporosis, either yearly intravenous zoledronic acid(23) or more frequent doses of oral alendronate or risedronate.(24,25) One limitation to systemic markers of bone remodeling is their lack of site specificity, because for a given biomarker level, the rate of bone remodeling assessed histologically can differ 10-fold across various skeletal sites.(26) Histologic assessment of bone remodeling has been conducted in several preclinical and clinical studies associated with BP treatment for osteoporosis, yet few data exist for BP doses used in cancer treatment.

The objective of this study was to determine the effect of cancer doses of zoledronic acid administered as monthly intravenous infusions on tissue-level bone remodeling using a beagle dog model. Specifically, our focus was on change to intracortical bone remodeling of the mandible, although other skeletal sites, namely, the rib and the tibia, also were examined. As a comparison to the cancer dose of zoledronic acid, additional animals were treated with a daily oral dose of alendronate to mimic treatment used for postmenopausal osteoporosis. Our hypothesis was that monthly intravenous zoledronic acid would significantly suppress intracortical remodeling at all skeletal sites compared with both vehicle- and alendronate-treated animals.

## Methods

### Animals

All experimental procedures were approved by the Indiana University School of Medicine Institutional Animal Care and Use Committee prior to the start of the study. Sixty skeletally mature female beagles (1 to 2 years old) were purchased from Marshall Farms USA (North Rose, NY). Animals were housed two per cage in environmentally controlled rooms at Indiana University School of Medicine's Association for Assessment and Accreditation of Laboratory Animal Care (AAALAC)–accredited facility.

### Experimental design

Following 1 week of acclimatization, animals were assigned to one of three treatment groups (*n* = 20/treatment). Dogs were treated with either monthly intravenous infusion of vehicle (VEH, saline) or zoledronic acid (ZOL) or with daily oral dosing of alendronate (ALN, Sigma-Aldrich). ZOL was administered at a dose of 0.067 mg/kg, which corresponds to the 4 mg dose used in cancer patients adjusted on a milligram per kilogram basis.(27) Zoledronic acid dissolved in saline (ZOL) or saline alone (VEH) was administered in a 40 mL volume via an over-the-needle catheter (20-guage) in the cephalic vein. Infusions took place over a 15-minute period in accordance with previously published protocols.(28) For intravenous infusions of ZOL or VEH, animals were sedated using 0.15 mL Domitor (medetomidine, 1 mg/mL), which then was reversed with 0.2 mL Antisedan IM (atipamezole, 5 mg/mL) at the end of the infusion period. ALN was administered at a dose of 0.20 mg/kg per day, equivalent (on a milligram per kilogram basis) to the 10 mg dose used for postmenopausal osteoporosis.(29,30) This dose has been shown previously to significantly suppress trabecular bone remodeling in a beagle dog model by 71% within a year.(31) ALN was dissolved in saline and administered to the dogs orally with a syringe each morning after an overnight fast and at least 2 hours prior to feeding.

Animals in the three groups were treated for either 3 months (*n* = 10/treatment) or 6 months (*n* = 10/treatment). Prior to necropsy, animals were injected with calcein (0.20 mL/kg IV) using a 2-12-2-5 labeling schedule, meaning that label was injected on 2 consecutive days, 12 days were allowed to pass, another 2 consecutive days of label were given, and then animals were euthanized 5 days later Animals were euthanized by intravenous administration of sodium pentobarbital (Beuthanasia-D Special, 0.22 mg/kg). After death, the right hemimandbile, right ninth rib, and right tibia were dissected free and stored in 70% ethanol.

### Histologic processing

**Fig. 1:**
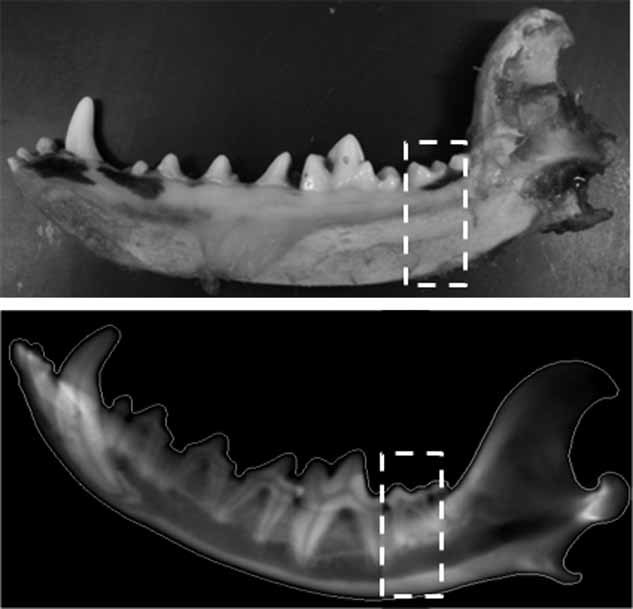
Photograph and DXA images depicting the second molar region, at which dynamic histologic analyses were conducted.

A portion of the mandible (∼5 mm) near the second molar region (Fig. 1) was segmented by making parallel buccal-lingual cuts using a band saw with a diamond-coated blade while under constant irrigation. A 5 mm segment of the rib (located at the spot of greatest curvature) and a 5 mm segment of the distal tibia (4 cm proximal to the distal end) also were prepared.

Tissues were stained with basic fuchsin in order to assess bone matrix necrosis.(17) Using 1% basic fuchsin dissolved in increasing concentrations of ethanol, specimens were stained according to the following schedule: 48 hours in 80% (with one change to fresh 80% solution after 24 hours), 48 hours in 95% (with one change to fresh 95% solution after 24 hours), and 48 hours in 100% (with one change to fresh 100% solution after 24 hours). Bones were placed under vacuum (20 in Hg) for all stages. Calcein labeling can be clearly observed in fuchsin-stained tissue.(17)

Following basic fuchsin staining, bones were washed in 100% ethanol for 10 minutes and then placed in 100% methyl methacrylate (MMA, Aldrich). Specimens then were transferred to a solution of MMA + 3% dibutyl phthalate (DBP, Sigma-Aldrich) for 3 to 7 days under vacuum and then embedded using MMA + DBP + 0.25% catalyst (Perkadox 16^3^, Akzo Nobel Chemicals). Sections (80 to 100 µm) from each bone segment were cut in a cross-sectional plane by making parallel buccal-lingual cuts using a diamond wire saw (Histosaw, Delaware Diamond Knives).

### Histologic assessment

Histologic measurements were made using a semiautomatic analysis system (Bioquant OSTEO 7.20.10, Bioquant Image Analysis Co.) attached to a microscope (Nikon Optiphot 2 microscope, Nikon) with a fluorescent light source. For most skeletal sites, a single cross section was assessed for intracortical bone formation rate. One exception was the 6-month ZOL-treated animals, in which in the assessment of a single mandible section from the 10 animals, only one osteon was found. Three additional sections, one additional section from the second molar region and two sections from the fourth premolar region of the mandible, were analyzed in these animals to increase the sampling region; therefore, data for the 6-month ZOL-treated mandible represent four sections. We also assessed a second region of the mandible (near the fourth premolar) of 6-month VEH- and ALN-treated animals to determine if changes at the second molar region were representative of the mandible at large. All slides were blinded to treatment during analyses. For mandible sections, data were collected separately for alveolar bone regions (defined as bone above the most distally observed portion of the tooth root) and nonalveolar bone regions (the remainder of the tissue).(17) The cortical bone of the entire cross section of the rib and tibia was assessed. Under ultraviolet light, the bone area (B.Ar.), number of labeled osteons (L.Os.#, osteons with either single or double label), the total length of osteonal labeled surface (L.S.), and the mean interlabel distance (Ir.L.Dis.) were measured. For L.S., all label within osteons was measured such that if an osteon had double label, the length of each was measured. Mineral apposition rate (MAR, µm/day) was calculated as Ir.L.Dis./12, where 12 is the number of days between labels. Intracortical bone formation rate (%/year) was calculated as [MAR × (L.S./2)/B.Ar. × 100] × 365. If a particular site for a given animal had single-labeled osteons but no double-labeled osteons, a value of 0.3 was used for MAR.(32) If no label was present, indicative of no active formation during the period of assessment, MAR was considered to be a missing value. In these cases, rather than use a missing value for bone formation rate (BFR), which necessitates MAR for calculation, we have considered BFR to be 0 so as to reflect the absence of bone formation activity. All measures and calculations were in accordance with American Society for Bone and Mineral Research (ASBMR)–recommended standards.(33)

Bone matrix necrosis in the mandible was assessed by bright-field microscopy, as described previously.(17) Regions of bone void of basic fuchsin stain larger than 500 µm^2^ were considered necrotic. For all animals, four complete cross sections of the mandible from two different regions (second molar and fourth premolar) were assessed for matrix necrosis.

### Statistics

Statistical tests were performed using SAS software (SAS Institute, Inc.). Differences among the three treatment groups within each time point (3 or 6 months) were evaluated using a one-way analysis of variance (ANOVA). When a significant overall *F* value (*p* < .05) was noted, differences between individual group means were compared using Fisher's protected least-significant-difference (PLSD) post hoc test. To determine the relative effect of ZOL across the four skeletal sites (i.e., alveolar mandible, nonalveolar mandible, rib, and tibia), absolute and percent differences in BFR for each ZOL-treated animal were calculated using the average values of the VEH animals at each site. This was done separately for the 3- and 6-month time points. These absolute and percent differences were compared across skeletal sites using a one-way ANOVA with repeated measures, with PLSD post hoc tests used to compare individual group means. For all tests, *p* ≤ .05 was considered significant.

## Results

There was no difference among group body masses at baseline or at the conclusion of the 3 or 6 months of treatment. All animals completed the 3 or 6 months of treatment without complication, and there was no evidence of oral lesions in any dog during the study. There was no evidence of any bone matrix necrosis, assessed by basic fuchsin staining, in the mandibles of any animal after either 3 or 6 months of treatment.

**Fig. 2:**
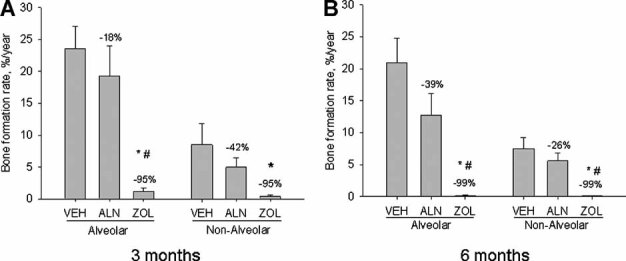
Intracortical bone formation rate of the mandible. (*A*) After 3 months, animals treated with monthly intravenous zoledronic acid (ZOL) had significantly a lower BFR in the alveolar and nonalveolar regions of the mandible compared with vehicle-treated controls (VEH); alveolar rates in ZOL-treated animals also were significantly lower than in alendronate-treated animals (ALN). (*B*) After 6 months, ZOL treatment produced near-complete suppression of bone formation rate in both alveolar and nonalveolar regions. Values above bars represent percentage of value compared with VEH-treated animals within time point and region. *p* < .05 versus VEH (*) or ALN (#). Data presented as mean ± SE.

Following 3 months of treatment, ZOL significantly reduced intracortical bone remodeling of the mandible (Fig. 2*A*). Compared with VEH, ZOL-treated animals had 95% lower intracortical BFR in both the alveolar and nonalveolar portions of the mandible (both *p* < .05). This lower turnover rate with ZOL treatment was the result of fewer active sites (labeled osteons) and a lower MAR compared with VEH (Table 1). ALN did not significantly suppress intracortical BFR, labeled osteon number, or MAR in either region of the mandible compared with VEH.

**Table 1:** Intracortical Turnover Properties of Mandible, Rib, and Tibia

	3 months			6 months		
		
	VEH	ALN	ZOL	VEH	ALN	ZOL
Alveolar Mandible						
Labeled osteon number, #/mm^2^	1.33 ± 0.31	0.88 ± 0.25	0.10 ± 0.04*#	0.89 ± 0.12	0.65 ± 0.17	0.009 ± 0.004*#
Mineral apposition rate, µm/day	2.16 ± 0.16	1.97 ± 0.09	1.38 ± 0.23*#	2.24 ± 0.16	1.78 ± 0.32	0.30 ± 0*#
Non-Alveolar Mandible						
Labeled osteon number, #/mm^2^	0.50 ± 0.13	0.46 ± 0.09	0.09 ± 0.05*#	0.46 ± 0.07	0.39 ± 0.13	0.014 ± 0.005*#
Mineral apposition rate, µm/day	1.97 ± 0.08	1.91 ± 0.18	1.05 ± 0.25*#	1.81 ± 0.26	1.81 ± 0.21	0.30 ± 0*#
Rib						
Labeled osteon number, #/mm^2^	2.23 ± 0.56	2.45 ± 0.41	0.58 ± 0.13*#	1.94 ± 0.39	1.04 ± 0.19*	0.35 ± 0.12*
Mineral apposition rate, µm/day	1.24 ± 0.14	1.45 ± 0.06	0.83 ± 0.19#	1.68 ± 0.11	1.26 ± 0.29	1.08 ± 0.28*
Tibia						
Labeled osteon number, #/mm^2^	0.20 ± 0.07	0.23 ± 0.09	0.04 ± 0.01	0.22 ± 0.05	0.22 ± 0.07	0.07 ± 0.04
Mineral apposition rate, µm/day	1.15 ± 0.24	1.95 ± 1.06	1.04 ± 0.25	1.56 ± 0.18	1.31 ± 0.27	1.09 ± 0.33

Data presented as mean ± SE.

* *p* < 0.05 versus VEH.

# *p* < 0.05 versus ALN.

After 6 months of treatment, ZOL-treated animals had near-complete suppression of BFR in the alveolar (−99%) and nonalveolar (−99%) mandible compared with VEH-treated controls (see Fig. 2*B*). As with the 3-month animals, this lower turnover rate after 6 months of ZOL treatment was the result of fewer active sites (labeled osteons) and a lower MAR compared with VEH (see Table 1). ALN-treated animals did not differ from VEH-treated animals for any of the mandible parameters at 6 months.

Analyses of a second region of the mandible (fourth premolar) in 6-month VEH- and ALN-treated animals were consistent with our analyses of the second molar region described earlier, both in absolute terms and in comparisons among groups. In the second molar region, 6-month VEH-treated animals had a mean alveolar mandibular MAR of 2.24 µm/day and a mean alveolar BFR of 20.9%/year (see Fig. 2*B* and Table 1). At the fourth premolar region, the mean alveolar MAR was 2.18 µm/day, and the mean alveolar BFR was 19.1%/year in VEH-treated animals; this was not significantly different from the second molar region (*p* > .50). Furthermore, the effect of ALN also was consistent between the two regions of the mandible, with the second molar region showing a 21% lower MAR and 39% lower BFR with ALN treatment compared with VEH (see Fig. 2*B* and Table 1); the fourth premolar region showed an 18% lower MAR and 43% lower BFR with ALN treatment.

**Fig. 3:**
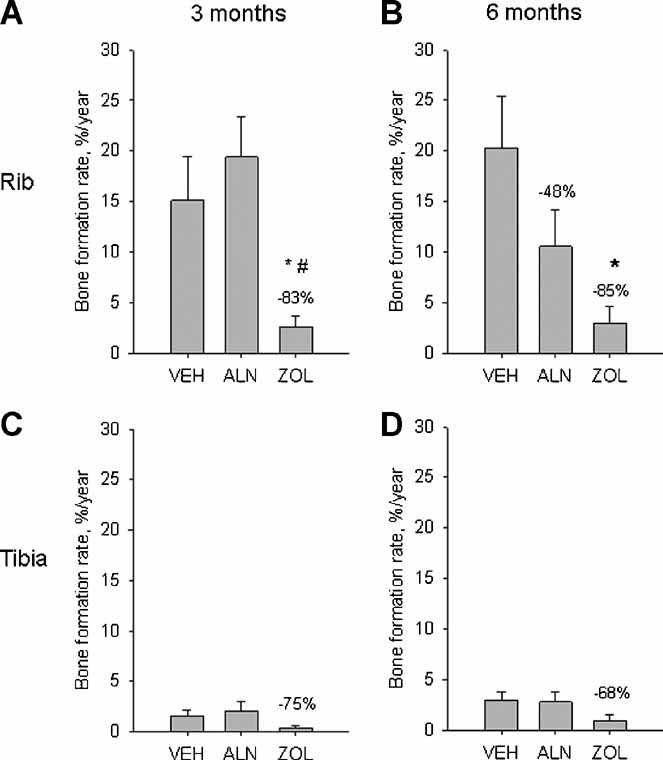
Intracortical bone formation rate of the rib (*A, B*) and tibia (*C, D*). (*A*) After 3 months, animals treated with monthly intravenous zoledronic acid (ZOL) had a significantly lower BFR in the rib compared with vehicle- (VEH) and alendronate-treated (ALN) animals. (*B*) After 6 months, animals treated with ZOL were significantly lower than VEH. (*C, D*). There was no significant effect of ZOL or ALN treatments on intracortical BFR of the tibia compared with VEH. Values above bars represent percentage of value compared with VEH-treated animals within time point and region. *p* < .05 versus VEH (*) or ALN (#). Data presented as mean ± SE.

The effects of ZOL treatment on the rib were consistent with those of the mandible. Compared with VEH, ZOL significantly suppressed intracortical BFR by 83% (3 months) and 85% (6 months) (Fig. 3*A,B*). This lower BFR was the result of fewer active sites (labeled osteons) and MAR compared with VEH at both time points (see Table 1). ALN did not significantly alter intracortical BFR, labeled osteon number, or MAR of the rib at 3 months but resulted in significantly fewer labeled osteons (−46%) compared with VEH by 6 months. Tibial intracortical BFR was nonsignificantly lower at 3 months (−75%) and 6 months (−68%) in ZOL-treated animals compared with VEH (Fig. 3*C,D*), whereas there was no effect of ALN at this site (see Fig. 3 and Table 1).

**Fig. 4:**
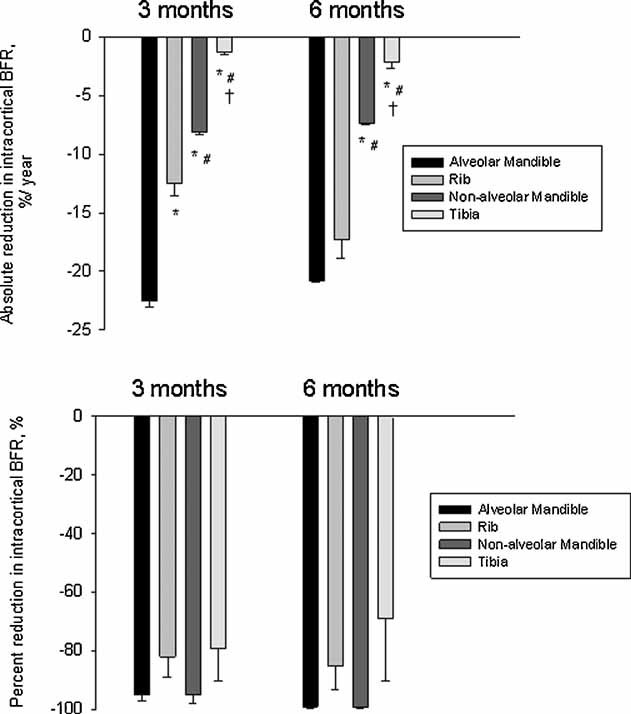
Comparison of ZOL effect on intracortical remodeling suppression across the various bone sites of assessment. At both 3 and 6 months, the absolute reduction in intracortical BFR was determined relative to VEH-treated animals for the alveolar mandible, rib, nonalveolar mandible, and tibia. Additionally, the percent reduction in intracortical BFR was compared among the four sites at 3 and 6 months. Values represent mean ± SE of the reduction in BFR compared with VEH. *p* < .05 versus alveolar mandible (*), rib (#), and nonalveolar mandible (†).

The absolute suppression of intracortical bone formation rate with ZOL treatment significantly differed among the four skeletal sites (Fig. 4). After 3 months of ZOL treatment, the absolute reduction in BFR compared with VEH treatment was significantly different at each of the four sites, with the effect at the alveolar mandible > rib > nonalveolar mandible > tibia. After 6 months of ZOL treatment, there was a nonsignificant difference in the suppression of intracortical BFR in the alveolar mandible and rib (*p* = .06), whereas both those sites had significantly greater suppression of remodeling than the nonalveolar mandible and tibia. When expressed at a percentage of VEH-treated animals, the effect of ZOL did not significantly differ among the four sites at 3 months (*p* = .23) or 6 months (*p* = .20).

## Discussion

In humans and other large animal species, cortical bone undergoes remodeling within the cortex.(34) Intracortical remodeling, similar to remodeling on bone surfaces, serves to renew bone tissue that contains microdamage or that becomes nonviable.(35,36) We and others have documented the remodeling suppressive effects of BP treatment on intracortical remodeling at various skeletal sites,(26,37–42) including the mandible.(17) Following 3 years of daily oral alendronate, at a dose five times higher than that used in the current study, intracortical bone formation in the alveolar bone of the mandible in beagle dogs was significantly lower (−84%) than in vehicle-treated animals.(17) At a dose consistent with that used clinically for osteoporosis treatment and identical to that used in the current report, alveolar remodeling was nonsignificantly lower than vehicle (−67%) after 3 years.(17) These previous results, combined with the current study, illustrate that suppression of mandibular remodeling with osteoporosis dosing regimens (specifically daily oral alendronate) is modest and has a relatively slow onset. Conversely, the changes to intracortical bone remodeling with intravenous zoledronic acid, at doses consistent with those used in cancer patients, are severe and rapid. Mandibular remodeling was suppressed by 95% after 3 months and 99% after 6 months of zoledronic acid. These data highlight distinct differences in tissue-level remodeling suppression of the mandible (as well as the rib and tibia) resulting from BP treatment regimens used for osteoporosis (daily oral alendronate) and cancer (monthly intravenous zoledronic acid).

The mechanism for differences in the remodeling suppression profiles of these two treatment regimens are likely multifactorial. The binding affinity of zoledronic acid is higher than that of alendronate, meaning that zoledronic acid has a greater attraction for and stronger attachment to mineral surfaces.(43,44) This factor alone, however, is unlikely to account for the dramatic differences noted in this study. More likely it is the combination of this higher affinity coupled with the dosing amount and route that account for the effects of zoledronic acid on remodeling suppression. There is a linear relationship between BP dose and skeletal uptake,(45) suggesting that cumulative dose could have a significant impact on the amount of drug to which the skeleton is exposed. Additionally, skeletal uptake of BP is significantly higher with intravenous dosing compared with oral dosing, even when differences in bioavailability profiles between the two routes are matched.(46) These differences in mineral affinity and skeletal uptake may explain the differential remodeling suppression profiles with osteoporosis treatment versus cancer treatment regimens.

While there was near-complete remodeling suppression of the mandible with intravenous ZOL (reduced from >20%/year to <0.1%/year), there remained measurable amounts of bone remodeling at other cortical sites. Comparison of the effects of ZOL on BFR across the four skeletal sites assessed show significant site-specific effects on the absolute level of intracortical bone formation suppression (Fig. 4). The effect of ZOL is greatest in the alveolar portion of the mandible, followed in order by the rib, nonalveolar mandible, and tibia. The magnitude of effect, on an absolute basis, appears intimately tied to the level of turnover in the untreated condition because the BFR in VEH animals showed alveolar mandible > rib > nonalveolar mandible > tibia. This is consistent with previous analyses in our lab, which showed that the absolute level of turnover suppression with BP treatment is related to the basal turnover rate.(47)

These data highlight the need for caution in generalizing changes in bone remodeling observed using systemic biomarkers. Clinical studies have shown similar degrees of remodeling suppression with BP regimens used for cancer(19–22) and osteoporosis.(23–25) Systemic biomarkers were not measured in the current study, yet previous reports using doses of intravenous zoledronic acid that were four times higher than those used in this study have shown suppression of urine cross-linked N-telopeptide of type I collagen (NTX) by approximately 75% in both normal and tumor-bearing dogs.(28,48) Assuming that biomarker levels of remodeling would be similar in the current ZOL-treated animals, this emphasizes that such biomarker measures, while quite useful on a systemic basis, have limitations with respect to knowing effects of treatment on specific skeletal sites. Given that the percent reduction in BFR with ZOL among the skeletal sites was not significantly different (see Fig. 4), it is possible that histologic measures of bone remodeling at one site, such as the iliac crest, can give some insight into the magnitude of effect throughout the skeleton.

The differential effects of remodeling suppression between BP dosing for osteoporosis and cancer have clear relevance for BP-related osteonecrosis of the jaw (BRONJ). The risk of BRONJ is significantly greater and the mean time to onset is shorter in cancer patients compared with those treated with these drugs for osteoporosis.(5) The current report is consistent with these clinical data, showing that following the initiation of treatment (3 to 6 months), the level of remodeling suppression is more severe with intravenous zoledronic acid than with oral alendronate. Whether or not the speed of onset or degree of remodeling suppression plays a role in the pathophysiology of BRONJ remains to be determined.

We have shown previously that suppression of intracortical remodeling following 3 years of oral alendronate treatment is associated with the accumulation of nonviable bone matrix, defined as an absence of osteocytes and nonpatent canaliculi.(17) This finding has led us to hypothesize that focal regions of osteocytes become nonviable, and these regions are unable to be remodeled sufficiently owing to the effects of BPs.(17) There were no regions of bone matrix necrosis in the mandibles of any animals in this study. This suggests, not unexpectedly, that the development of these regions, which involves filling in of the canalicular network with mineral, takes time. Thus, even though remodeling of the mandible is significantly suppressed within the first 3 months with zoledronate at cancer doses, it appears to take greater than 6 months for the development of matrix necrosis.

Recently, we have reported that trabecular bone appears to have a lower limit of suppression with BP treatment,(47) interpreted as evidence against the theory of remodeling oversuppression.(49,50) Current data suggest that our earlier report may be limited to trabecular bone or to alendronate treatment because the level of intracortical remodeling suppression with intravenous ZOL clearly can reach a nadir at or close to zero at some skeletal sites. The dramatic effects of cancer treatment doses of zoledronic acid on bone remodeling in the mandible suggest the need to consider alternative doses or dosing regimens in cancer patients. Clearly, the current study does not address the level of remodeling that is necessary for the primary goal of such treatment regimens—to offset adverse changes associated with the cancer itself. It is possible that complete suppression of mandibular remodeling may be a necessary consequence of successfully controlling the cancer-induced changes. However, early clinical data suggest that such large doses are not necessary for equivalent suppression of remodeling, as assessed by biomarkers. Early work aimed at controlling hypercalcemia of malignancy concluded that of several zoledronic acid doses (0.002, 0.005, 0.01, 0.02, and 0.04 mg/kg), the highest two doses (equating to 1.2 and 2.4 mg, respectively, for a 60 mg person) provided superior control of serum calcium compared with the others.(51) Two subsequent studies in which patients were treated with a single intravenous dose of zoledronic acid (1, 2, 4, 8, or 16 mg)(52) or monthly intravenous zoledronic acid (0.1, 0.2, 0.4, 0.8, 1.5, 2.4, or 8 mg)(19) reached the conclusion that doses of less than 1 mg were inferior for suppression of remodeling biomarkers, yet doses between 1.5 and 16 mg were all comparable. While further studies showed that doses above 4 mg result in renal safety issues,(20) very little work has been done on doses lower than 4 mg despite the fact that these data suggest the half this dose may be effective in this patient population.

In conclusion, we show significant intracortical remodeling suppression resulting from monthly intravenous zoledronic acid dosing regimens analogous to those used in cancer patients. These changes are more pronounced than those that occur with dosing regimens used for osteoporosis (daily oral alendronate). Most notable was the suppression imparted by zoledronic acid on the mandible, where remodeling suppression was near complete.
